# YXQN Reduces Alzheimer’s Disease-Like Pathology and Cognitive Decline in APPswePS1dE9 Transgenic Mice

**DOI:** 10.3389/fnagi.2017.00157

**Published:** 2017-05-23

**Authors:** Xiaowan Wang, Runmin Song, Wenliang Lu, Ziyu Liu, Lichun Wang, Xiaojuan Zhu, Yanjun Liu, Zijie Sun, Jiang Li, Xiaomeng Li

**Affiliations:** ^1^The Key Laboratory of Molecular Epigenetics of MOE, Institute of Genetics and Cytology, Northeast Normal UniversityChangchun, China; ^2^School of Traditional Chinese Pharmacology, Tianjin University of Traditional Chinese Medicine, TianjinChina; ^3^Division of Endocrinology, Metabolism and Molecular Medicine, UCLA School of Medicine, Charles R. Drew University of Medicine and Science, Los AngelesCA, United States; ^4^Department of Genetics, Stanford University School of Medicine, StanfordCA, United States; ^5^Dental Hospital, Jilin University, ChangchunChina

**Keywords:** Yangxue Qingnao, Alzheimer’s disease, APP/PS1 mice, amyloid-β, APP processing

## Abstract

Alzheimer’s disease (AD) is the world’s most common form of dementia, in which aggregation of amyloid-β (Aβ) is the hallmark. Unfortunately, few medicines have succeeded to completely cure AD. Yangxue Qingnao (YXQN) is a Chinese traditional medicine, and its pharmacological effect is improving cerebral blood flow. In this study, we firstly demonstrated that YXQN reduced AD-like pathology and cognitive impairment in APPswePS1dE9 (APP/PS1) mice with 2 months administration. Our data showed that YXQN substantially ameliorated behavioral defects in 10-month old APP/PS1 mice using Morris Water Maze and Y-maze tests, in which the cognitive ability of YXQN high-dose group approaches to wild type mice. Next, we focused on the brain pathological alterations in the YXQN group by three experiments, including thioflavin-S, congo-red, and Aβ-immunohistochemistry staining. The results demonstrated that the high-dose of YXQN dramatically suppressed amyloid plaques in the hippocampus and cortex of APP/PS1 mice, which showed a 47–72% reduction in plaque deposits, relative to the vehicle group. In addition, our data verified that YXQN decreased the cerebral amyloid load by attenuating β-secretase BACE1 and γ-secretase PS1 in the pathological processing of APP, and promoting the level of α-secretase ADAM10 in the physiological processing of APP to generate more sAPPα, which combats amyloidosis formation, and also carries out neurotropic and neuroprotective effect. Taken together, our results strongly suggest that YXQN could be a potential medicine for AD, and provide new evidence for further AD drug research and development.

## Introduction

Alzheimer’s disease (AD) accounts for a large number of dementia cases and afflicts more than 48 million individuals worldwide (Alzheimer’s Association, 2015). It is a degenerative disease of the central nervous system, with amyloid-β (Aβ) deposition in the brain as a crucial pathological hallmark (Campion et al., 2016), which antedates any other triggered pathological changes of AD, as has been demonstrated by positron emission tomography (PET) (Forsberg et al., 2008; Brendel et al., 2015). Unfortunately, all the medicines designed to prevent the production and aggregation of Aβ have invariably failed in their clinical III trials, including the most anticipated BACE1 inhibitor and Aβ monoclonal antibody.

As an AD triggering molecule, Aβ is a proteolytic product of the amyloid precursor protein (APP) via the amyloidogenic pathway, in which APP is cleaved by β-secretase (BACE1) to produce extracellular release part-soluble APP peptide-β (sAPPβ), and C-terminal fragment-β (CTFβ)—also known as C99. Subsequently, cleavage of C99 by γ-secretase complex [mainly presenilin 1 (PS1)] releases Aβ, mainly Aβ42 and Aβ40 (Oddo et al., 2004; Dong et al., 2006). While, in the dominant pathway of APP (non-amyloidogenic pathway) under normal conditions *in vivo*, initial processing of APP by α-secretase (ADAM10) within the Aβ domain generates a secreted form of sAPPα and CTFα (C83), which in turn precludes Aβ generation (Kim and Tsai, 2009; Portelius et al., 2011). In addition, sAPPα provides neuroprotection and promotes neuron outgrowth (Pimplikar and Ghosal, 2011; Milosch et al., 2014).

For AD research and medicine development, APPswe/PS1dE9 double transgenic mice (APP/PS1 for short) expressing a chimeric mouse/human APP695 with the Swedish mutation (KM594/595NL), together with a mutant human PS1 protein with E9 deletion were constructed by Jankowsky’s lab (Jankowsky et al., 2001). Compared with WT-APP, APPswe is more easily cleaved by BACE1, and PS1-dE9 shows higher γ-secretase activity to generate Aβ (Li et al., 2016). The APP/PS1 mice develop amyloid pathology in the brain at an age of 6–7 months and closely recapitulate the pathological characteristics and progressive course of AD (Perez et al., 2005; Puig et al., 2016).

Recent data showed that augmentation of cerebral blood flow (CBF) could be a new approach to the treatment of Alzheimer’s disease (Goldsmith, 2011). Further, CBF measured by arterial spin labeling MRI was reported as a preclinical marker of Alzheimer’s disease (Wierenga et al., 2014). Yangxue Qingnao (YXQN) extract is a famous Chinese medicine to improve CBF and brain nourishment. It is composed of 11 Chinese herbs, and is a patent medicine used for alleviating headache and dizziness treatment in clinics for 20 years. Four of them come from Siwu Tang, Four Herbs Decoction, one of the most famous prescriptions for activating blood circulation, which is recorded in the Chinese first national pharmacopeia, Prescriptions People’s of the Welfare Pharmacy. The other seven herbs additionally activate blood circulation, and play the role of anti-oxidation, protection of neurons and regulation of the enzymes targeting the nervous system (Wang et al., 2013).

In this study, we explored the effects of YXQN extract on AD pathology and cognitive function in APP/PS1 transgenic mice. We assessed the amyloidosis changes by YXQN administration, and also detected the proteases involved in the proteolytic process of APP, including ADAM10, BACE1, and PS1, in order to develop the potential Chinese medicine for AD treatment.

## Materials and Methods

### Drug Supplementation

Yangxue Qingnao is a widely applied Chinese traditional patent medicine, consisting of 11 active components, including Angelica sinensis, Ligusticum chuanxiong hort, White peony root, Prepared radix rehmanniae, Uncaria, Spatholobus suberectus, Prunella vulgaris, Cassia seed, Nacre, Rhizoma corydalis, and Asarum, effects of which are shown in **Table 1**. YXQN extract was provided by the TIANJIN TASLY Pharmaceutical co., LTD, the processing of the product followed strict quality control, and the ingredients were subjected to standardization. YXQN extract was dissolved in distilled water at 0.069 g, 0.208 g, and 0.624 g per mL for use. Positive control Aricept donepezil (HCl salt) tablets (commonly referred to as donepezil; Eisai (China) Pharmaceutical co., LTD) was prepared with a concentration of 0.103 mg/mL for use. The diluted YXQN and donepezil were used for oral administration by 0.1 mL/10 g of weight in mice. In all, the drug dosages are YXQN low-dose at 0.69 g/kg, YXQN middle-dose at 2.08 g/kg, YXQN high-dose at 6.24 g/kg, and donepezil at 1.03 mg/kg. Besides, the dosages of YXQN middle-dose and donepezil are equal with clinical application doses for patients in Pharmacology.

**Table 1:** Characterization of the herbs included in YXQN.

Herbs	Percentage content (%)	Identified compounds	Effects
**Siwu Tang (Four herbs decoction)**			
Angelicae sinensis	6.76	Ferulic acid	**Increasing cerebral blood flow and improving blood circulation:** anti-oxidant, neuroprotection, anti-anginal, anti-apoptotic, synergistic promotion of blood vessel regeneration, decreasing the brain infarct size, inhibiting neutrophil adhesion to endothelial cells, protective effect of vascular dysfunction and hypertension, maintaining blood-brain barrier integrity, et al. (Xu et al., 2009; Panth et al., 2016; Wang et al., 2016)
Ligusticum chuanxiong hort	6.76	Ligustrazine	
White peony root	5.41	Peoniflorin	
Prepared radix rehmanniae	5.41	Rehmannioside	
**The seven modified portion**			
Uncaria	13.51	Rhynchophylline	**Activating blood circulation, anti-oxidation, neuroprotection and enzyme regulation:** preventing neurotoxicity during ischemia, blocking of calcium channel, attenuating oxidative stress and neuronal damage, inhibiting the production of nitric oxide, radical scavenging effect, inhibiting myocardial infarction, up-regulation of Bcl-2, dopaminergic antagonist, inhibiting histamine release, anti-inflammatory, attenuates pro-inflammatory responses through down-regulation of MAPK/NF-κB signaling pathways, et al.(Xu et al., 2009; Song et al., 2012; Huang et al., 2014; Miao et al., 2016)
Spatholobus suberectus	13.51	Genistein	
Prunella vulgaris	13.51	Ursolic acid and 2-alpha-hydroxyursolic acid	
Nacre	13.51	Water-soluble extract	
Cassia seed	13.51	Naphthopyrones and Alaternin	
Rhizoma corydalis	6.67	_L_-Tetrahydropalmatine	
Asarum	1.35	Methyleugenol	


### Animal Treatment and Experiment Schedule

Amyloid precursor protein/presenilin 1 mice (B6C3-Tg (APPswe, PSEN1dE9) 85Dbo/J) were purchased from the Model Animal Research Center of Nanjing University (Nanjing, China). This study was carried out in accordance with the recommendations of the Chinese Council on Animal Care Guidelines, the Model Animal Research Center of Nanjing University. The protocol was approved by the Model Animal Research Center of Nanjing University.

In the AD model of APP/PS1 mice, degenerate cognitive function and Aβ deposits could be observed at 6–7 months (Jankowsky et al., 2004). Thus, we used equal numbers of female and male APP/PS1 mice at age of 8 months to explore the effects of YXQN and assessed AD pathology at the age of 10 months after 2 months administration. After 1 week of acclimatization to the cages, the 8-month APP/PS1 mice were randomly divided into five groups (vehicle group, *n* = 16; YXQN low-dose group, *n* = 17; YXQN middle-dose group, *n* = 18; YXQN high-dose group, *n* = 18; donepezil group, *n* = 16) and then orally administered with attenuated donepezil, YXQN (low-, middle-, and high-dose), or water (0.1 mL/10 g weight) for 2 months. Littermates were used as WT vehicle control (*n* = 16) throughout the study and were given distilled water for 2 months as well. Mice were housed in standard laboratory cages with a 12 h light and dark cycle along with free access to food and water.

**FIGURE 1:**
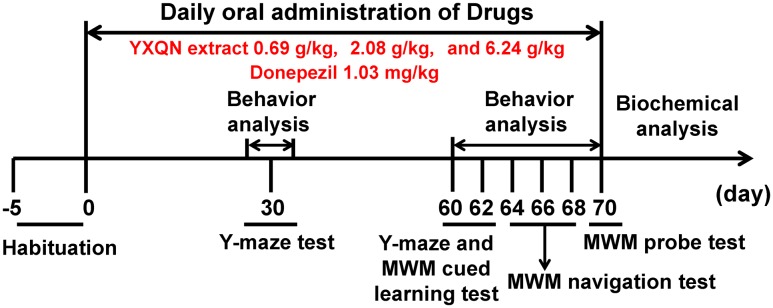
**Behavior and biochemical analysis schedule to study the effects of YXQN on APP/PS1 mice**.

The experimental design of behavior and biochemical analysis is shown in **Figure 1**. During the 2-month administration period, cognitive function of the five groups of APP/PS1 mice was measured by Y-maze at 30th and 60th day, and measured by Morris Water Maze (MWM) at the termination of drug supplementation. After that, a set of the biochemical index in the brain was investigated. Therefore, all AD pathological indices in this research were determined in 10-month-old APP/PS1 mice.

### Behavioral Assessments

The short-term spatial memory ability for five groups of APP/PS1 mice was tested by Y-maze spontaneous alternation. In brief, we placed mice separately into a radially symmetric Y-maze with three arms (arms: 40 cm long, 4 cm wide; walls: 30 cm tall). The number and sequence of arm entries were scored over 8 min. Alternations were calculated when a mouse consecutively traveled to all three arms in any order without re-entering the previous arms. Percent of alternation was formulated as the ratio of the number of alternations to the number of total arm entries minus two (Lalonde, 2002; Town et al., 2008).

The spatial learning-memory ability was assessed by the MWM tests, which consists of the orientation navigation tests and the spatial probe tests. Mainly, the MWM contains a circular tank (diameter: 120 cm) filled with water at 24°C and a hidden platform (diameter: 15 cm) positioned 1–2 cm below the opaque water in the middle of the northeast quadrant. Before the measurement, mice were trained to find the platform for 3 days, orienting by cues on the wall of the tank as spatial references (Vorhees and Williams, 2006). For orientation navigation tests, mice were allowed to search for the platform for 120 s and to stay on the submerged platform for 30 s, before they were placed back in the cage under a heater to dry. Mice were tested four times a day for six consecutive days. The escape latency and the swim path tracking until the mice landed on the platform were recorded on videotape. For the probe trials, which were performed to determine memory retention on the next day (day 7), the platform was removed, and mice were placed into the pool from the opposite quadrant where the platform had been located. They were allowed to swim for 120 s, and the number of platform crossings, the percent of time spent in each quadrant, and the swim path tracking were recorded on videotape.

### Histological Examinations

After behavioral analysis, mice were euthanized with pentobarbital sodium and fixed in 4% paraformaldehyde after myocardial perfusion. Brains were dissected and embedded in paraffin for preparing sagittal sections and further staining analysis. The sections from each mouse were separately stained according to the following procedures:

For thioflavin-S staining, brain sections were stained with 0.01% thioflavin-S in 50% ethanol, and following differentiation in 50% ethanol (Heneka et al., 2013). Then, stained sections were analyzed with the Hg-Lamp for fluorescence excitation.

Congophilic amyloid staining by congo-red conformed to standard protocols (Vom Berg et al., 2012). Briefly, sections were incubated with 0.5% congo-red in 80% methanol and 20% glycerol for 20 min, and following differentiation with 0.2% KOH in 80% ethanol. Subsequently, nuclei were stained blue with hematoxylin.

In addition, brain sections were subjected to EnVision system immunohistochemistry to detect Aβ, ADAM10, BACE1, and PS1. The primary antibody 6E10 (Covance) was used for assessing Aβ deposition at 1:500 dilution. Moreover, the specific antibodies ADAM10, BACE1, and PS1 (Bioss) antibodies were used for staining and analyzing the expression of ADAM10, BACE1, and PS1 at 1:200 dilution.

Finally, all stained sections were analyzed using a BX41 or IX71 microscope (Olympus) and collected using DP Controller software. We performed the quantitative assessment of three defined regions per mouse brain. Plaque number and staining area were calculated by Image-Pro Plus 6.0 software. The staining area fraction was determined by dividing total plaque area by the area of the microscopic field.

### Western Blot

The homogenized brain tissues were eluted by boiling in SDS-sample buffer. Then, we performed SDS-PAGE to assess brain proteins using the Bio-Rad mini gel system (Vom Berg et al., 2012). In particular, Aβ and APPct levels were measured with urea-based electrophoresis, and then transferred onto polyvinylidene difluoride (PVDF) membranes. The membranes were then probed with antibodies at the appropriate dilutions, including Aβ and full-length APP (6E10, Covance) and APPct (A8717, Sigma–Aldrich) at 1:1000 (Sarajarvi et al., 2009; Heneka et al., 2013), ADAM10 (Bioss), BACE1 (Bioss), and PS1 (Bioss) at 1:500. We used β-actin (Abcam) as internal controls and Image-Pro Plus software for densitometric analysis.

### ELISA for Human Aβ

For ELISA test of Aβ40 and Aβ42 peptides from soluble and insoluble fractions, we homogenized frozen cerebral hemispheres (150 mg/mL wet weight) in PBS containing 1% SDS with protease inhibitors (Roche), followed by centrifugation at 100,000 g for 60 min at 4°C (Kawarabayashi et al., 2001; Vom Berg et al., 2012). The supernatant was removed as the soluble fraction, and the pellets (insoluble fraction) were dissolved in 70% formic acid. Then the insoluble fraction was neutralized by 1 M Tris buffer (pH 11). We used the Human Amyloid-β (aa1-40) or (aa1-42) Quantikine ELISA Kit to analyze Aβ40 and Aβ42 according to the manufacturer’s instructions (R&D Systems).

### Statistical Analysis

Data presented as means ± standard errors of the mean (SEM). All quantitative results including histology staining, Western blot, ELISA, and behavioral tests were analyzed by ANOVA with Dunnet’s *post hoc*. All analyses were carried out using SPSS 17.0 statistics software (Chicago, IL, United States).

## Results

### YXQN Counteracts Cognitive Decline in APP/PS1 Mice

**FIGURE 2:**
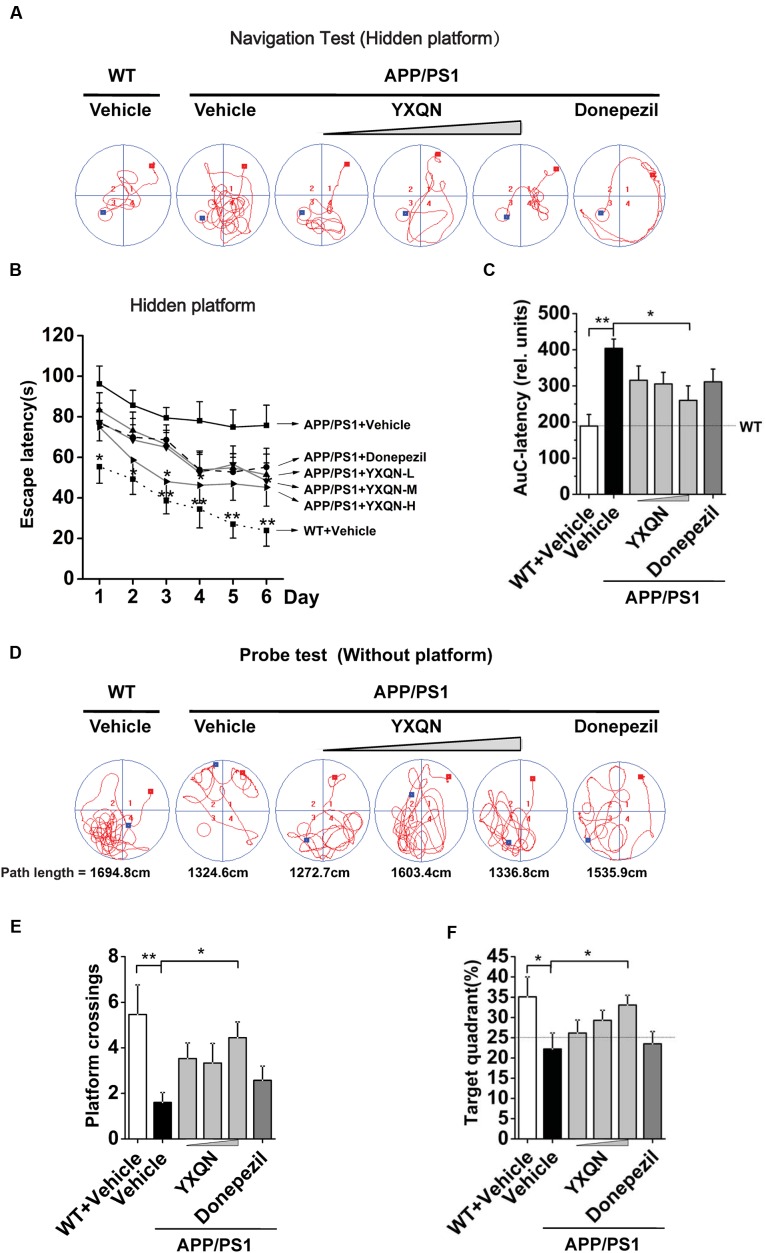
**Yangxue Qingnao improves the spatial and long-term memory decline in APP/PS1 mice by MWM.** **(A–C)** Navigation tests analysis in MWM by escape latencies for WT mice (*n* = 16) and the APP/PS1 mice with vehicle, YXQN extract or donepezil supplementation for 2 months (*n* = 14–17). **(A)** Representative path tracking in the navigation tests with hidden platform. **(B)** Average latencies curve of four trials per day in the six consecutive days. **(C)** The area under the curve (AuC) of the escape latency was calculated for each group for statistical comparison. **(D–F)** Probe tests analysis of individual groups utilized MWM on day 7. **(D)** Representative path tracking in the probe tests without hidden platform. **(E)** The average times that the mice crossed platform location in 120 s. **(F)** The percentage of searching time that the mice of individual groups spent in the target quadrant where the platform has been located in days 1–6. The drug dosages are YXQN low-dose (YXQN-L) at 0.69 g/kg, YXQN middle-dose (YXQN-M) at 2.08 g/kg, and YXQN high-dose (YXQN-H) at 6.24 g/kg. Data are represented as group mean ± S.E.M. All *post hoc* statistical comparisons are versus the vehicle APP/PS1 mice, *^∗^p* < 0.05 and *^∗∗^p* < 0.01 (Dunnet’s *post hoc*).

Utilizing 8 month old APP/PS1 mice and their littermates (WT), we evaluated the possible effects of YXQN on cognitive function of mice after 2 months of drug administration by two kinds of behavioral test, including MWM tests and Y-maze tests. Firstly, the APP/PS1 mice were administrated with vehicle, YXQN low-dose 0.69 g/kg, middle-dose 2.08 g/kg, and high-dose 6.24 g/kg (equivalent to 33, 100, and 300% clinical application dose), or donepezil 1.03 mg/kg (equivalent to 100% clinical application dose) per day from 8 to 10 months of age. At the time of termination of drug supplementation, mice were subjected to the navigation tests in the MWM to assess their spatial learning-memory formation. The search time to find the platform (escape latency) and path tracking were recorded. As shown in **Figure 2A**, compared with WT mice, the typical path tracking of the vehicle APP/PS1 mice was disorganized, indicating the mice searched for the hidden platform by a random trajectory. YXQN administration APP/PS1 mice showed shorter path lengths and selective search tracking, similar to WT mice. These data suggested an improvement in the spatial memory of AD mice from YXQN groups. Moreover, the average escape latencies of 6 consecutive days of each group were displayed in curves (**Figure 2B**). By statistics, escape latencies were demonstrated tend to decrease over time, and overall, latencies were significant different among groups (*F* = 23.475 day: *p* < 0.0001 group: *p* = 0.002; RM-ANOVA). The vehicle APP/PS1 showed the longest latencies, and the vehicle WT mice showed the shortest latencies on each day. Compared with vehicle AD mice, YXQN low-, middle-, and high-dose APP/PS1 mice and donepezil APP/PS1 mice showed different degrees of shortened latencies, and YXQN high-dose group most closely approached the latencies of the WT group, and presented significant differences with vehicle group on day 3 and 4 (*F* = 2.948 and 2.572; *p* = 0.036 and 0.036). Especially, on day 6, the latencies of YXQN low-, middle-, and high-dose APP/PS1 mice were remarkably shorter by 32.10, 36.06, and 40.32%, respectively, relative to vehicle APP/PS1mice. Next, for quantificational evaluation, the area under the escape latency curve were calculated, which represented the general cognitive level over six consecutive days. As shown in **Figure 2C**, compared to WT mice, the vehicle APP/PS1 mice presented a substantial increase in AuC-latency (*F* = 4.015; *p* < 0.0001), suggesting the declining spatial memory. The reduction in the escape latency was observed in the different dosages of YXQN and donepezil groups compared with the vehicle group. Particularly, the AuC-latency of the high-dose YXQN group showed the most significant decrease (*F* = 4.015; *p* = 0.011), approximating the AuC-latency of the WT group. Thus, YXQN, especially in high-dose, could substantially ameliorate the severe deficit in spatial and long-term memory formation in aged APP/PS1 mice.

The above results of the navigation tests with hidden platform were supported by a subsequent probe trial without the platform. Again, the typical path tracking of each group in 120 s was shown in **Figure 2D**. Within the similar total path length, the vehicle APP/PS1 mice swam randomly throughout the tank, indicating their poor memory retention of the location of the platform. However, the WT mice and also the YXQN administrated APP/PS1 mice used a spatially biased search strategy to locate the platform, indicating their good memory retention. Further, we calculated the platform location crossing times and the percent of target quadrant search time, both of which evinced the memory retention of the location where the hidden platform had been placed. The data suggested that there were more platform location crossing times and a higher percent of target quadrant search time in the YXQN high-dose group than the vehicle group in APP/PS1 mice (*F* = 2.300; *p* = 0.012; *F* = 2.779; *p* = 0.023). The platform location crossing times and percent of target quadrant search time of YXQN high-dose group were both approximate to the outcomes of WT group (*F* = 2.300; *p* = 0.001; *F* = 2.779; *p* = 0.010) (**Figures 2E,F**). These results provided evidence on the significant compensating effect of YXQN high-dose on cognitive deficits.

**FIGURE 3:**
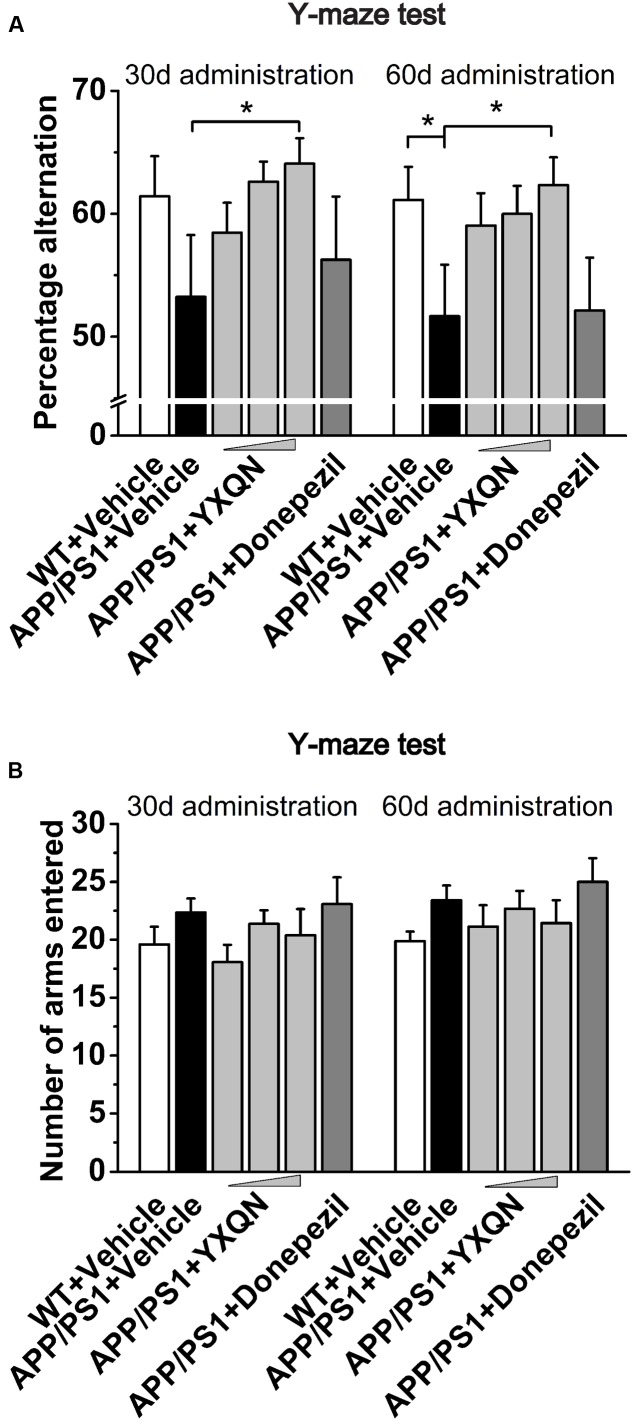
**Yangxue Qingnao improves the short-term spatial memory decline in APP/PS1 mice by Y-maze.** **(A)** Short-term spatial memory observed twice (administration 1- or 2-month) by percentage alternation among arms in WT mice (*n* = 16) and the APP/PS1 mice with vehicle, YXQN or donepezil administration (*n* = 14–17). **(B)** Total arm entries of individual groups. The drug dosages from low to high of YXQN extract are 0.69, 2.08, and 6.24 g/kg. Data are represented as group mean ± S.E.M. All *post hoc* statistical comparisons are versus the vehicle APP/PS1 mice, *^∗^p* < 0.05 (Dunnet’s *post hoc*).

To further confirm the above results, the Y-maze alternation tests were performed for the detection of short-term spatial memory ability. As shown in **Figure 3A**, compared with the vehicle APP/PS1 group, the percentage alternation of YXQN high-dose showed a notable increase in the memory test after 1 month of drug treatment (*F* = 1.490; *p* = 0.024). When 2 months of the drug treatment finished, again the YXQN high-dose group showed the most significant effect on increasing alternation (*F* = 2.183; *p* = 0.015), implying the enhancement of short-term memory by YXQN high-dose treatment. Further, in Y-maze alternation tests, the amelioration of cognitive ability was independent of motor ability (Reiserer et al., 2007), as manifested by the unchanged numbers of arms entered in each group (**Figure 3B**).

Above all, our results indicated that YXQN significantly improved cognitive deficits of APP/PS1 mice in a dose-dependent manner. Remarkably, the effect of decreased cognitive impairment was greater in the YXQN middle- and high-dose groups than the donepezil group. Intriguingly, with YXQN high-dose supplemented for 2 months, the spatial short- and long-term memory formation and retention of the APP/PS1 mice was similar to that of the littermates WT mice, suggesting that YXQN counteracts cognitive decline in APP/PS1 mice.

### YXQN Decreased Amyloid Burden in the Hippocampus and Cortex of APP/PS1 Mice

**FIGURE 4:**
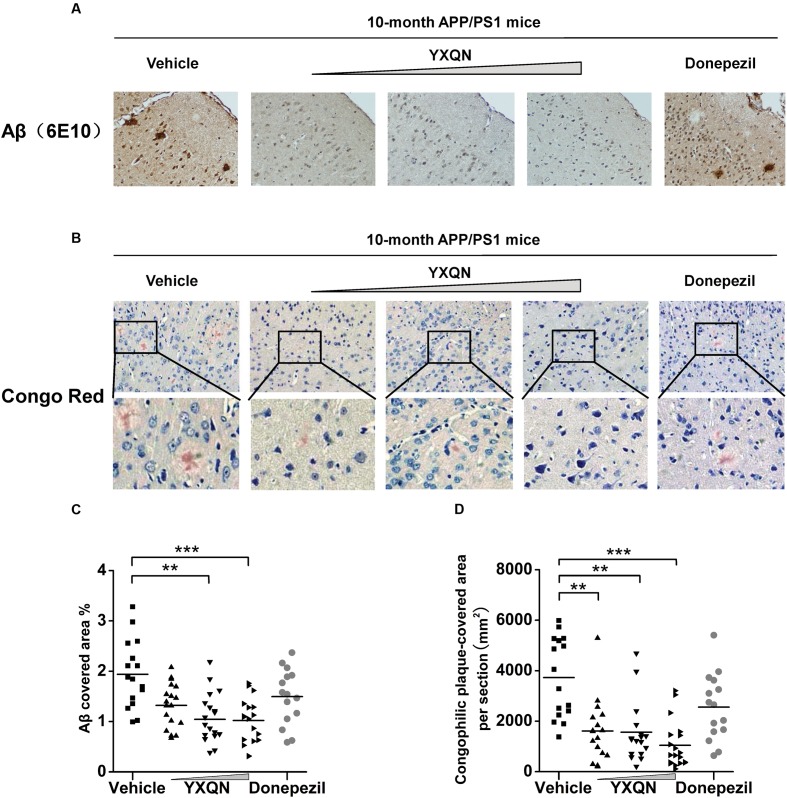
**Yangxue Qingnao reduces amyloid deposits in the brain of APP/PS1 mice.** **(A–D)** Aβ plaque load in 10-month-old APP/PS1 mice after 2 months of vehicle, YXQN, and donepezil administration assessed by Aβ reactive antibody 6E10 **(A,C)** or congo red **(B,D)**. **(A)** Analysis of Aβ deposition levels by immunohistochemical staining in APP/PS1 mice with vehicle, YXQN or donepezil supplementation (the percent of the cover area quantified in C, *n* = 15–18). **(B)** Congo red positive plaques in groups show representative cortical areas and higher-magnification images. **(D)** Quantification of the cover area of congophilic plaques (*n* = 15–18 mice per experiment group). The drug dosages from low to high of YXQN extract are 0.69, 2.08, and 6.24 g/kg. Mean ± S.E.M. For statistical analyses, one-way analysis of variance (ANOVA) was used **(C,D)**. *^∗^p* < 0.05, *^∗∗^p* < 0.01, and *^∗∗∗^p* < 0.0001.

Based on the fact that YXQN extract supplementation may ameliorate cognitive decline in the AD mouse model, we next investigated the core pathology of the AD-amyloid burden in APP/PS1 mice of each group. Firstly, using specific antibody 6E10, the Aβ plaques were stained in the sagittal brain sections of each group. As a result, substantial cerebral amyloidosis could be observed by Aβ-immunoreactive in vehicle APP/PS1 mice at 10 months of age (**Figure 4A**). However, YXQN low-, middle-, and high-dose groups all presented a forceful reduction in the Aβ deposition when compared with the vehicle group. The Aβ covered areas were reduced by approximately 50% in the APP/PS1 mice treated with the three dose levels of YXQN extract (*F* = 9.212; *p* < 0.0001 and *p* < 0.0001) (**Figure 4C**). Next, to corroborate the finding of the reduction of amyloid burden in YXQN groups, congo-red staining was performed, which specifically stains the amyloid plaque. Again, YXQN extract significantly decreased the Aβ burden in the brain of APP/PS1 mice (**Figure 4B**). Particularly, compared with the vehicle group, the YXQN high-dose group showed a 72% decrease in plaque covered area (*F* = 12.080; *p* = 0.002, *p* = 0.001, and *p* < 0.0001) (**Figure 4D**).

**FIGURE 5:**
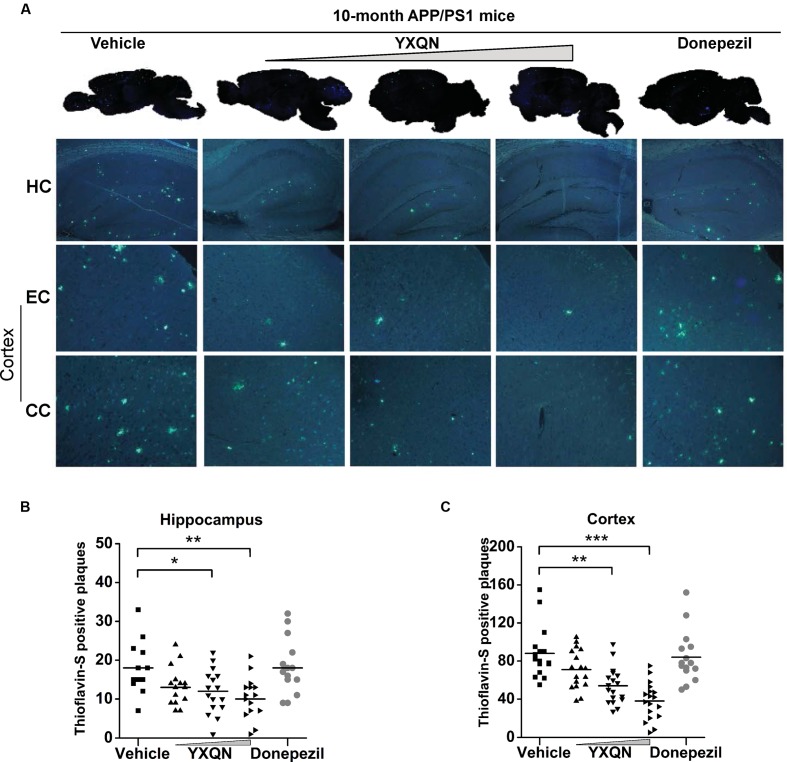
**Yangxue Qingnao decreases Aβ deposition that presents both in hippocampus and cortex of APP/PS1 mice.** **(A)** In the APP/PS1 mice with vehicle, YXQN, and donepezil supplementation, Aβ deposition was quantified in whole-brain sagittal plane and hippocampus (HC), entorhinal cortex (EC) or cingulate cortex (CC) area using thioflavin S. **(B,C)** Quantitative analysis of the number of Aβ plaques loaded in cortex region **(B)** and hippocampus region (**C**; *n* = 12–18). The drug dosages from low to high of YXQN extract are 0.69, 2.08, and 6.24 g/kg. Mean ± S.E.M. ANOVA with Dunnet’s *post hoc* analysis was used. *^∗^p* < 0.05, *^∗∗^p* < 0.01, and ^∗∗∗^*p* < 0.0001.

The hippocampus (HC), entorhinal cortex (EC), and cingulate cortex (CC) form the CC-EC-hippocampus, which is the most important element in a brain for organizing spatial memory and transforming short-term memory to long-term memory. This region is the first area to suffer damage in the brain of AD patients and is closely associated with neurodegeneration (Khan et al., 2014; Lopez et al., 2014; Chang et al., 2016). We therefore performed thioflavin-S staining on the brain sections to investigate whether YXQN administration alters Aβ deposition in the hippocampus and cortical areas, including the EC and the CC. Thioflavin-S is a kind of fluorochrome specifically binding to amyloid deposits, and can be excited to produce green fluorescence. As shown in **Figure 5A**, the YXQN middle- and high-dose groups both significantly decreased the amount of thioflavin-S positive plaques in the hippocampus, EC, and CC, compared with the vehicle group. By quantifying, YXQN middle- and high-dose groups, respectively, reduced the Aβ plaques by 32 and 44% in hippocampus areas (*F* = 5.938; *p* = 0.028 and *p* = 0.002) (**Figure 5B**), and also by 39 and 57% in the cortex areas, relative to the vehicle group (*F* = 15.051; *p* = 0.003 and *p* < 0.0001) (**Figure 5C**). Taken together, YXQN treatment groups had substantially reduced Aβ deposition in the hippocampus and cortical regions in a dose-dependent manner, suggesting that YXQN extract ameliorated cognitive impairment in MWM and Y-maze probably through reducing the Aβ deposition in the hippocampus and cortical areas of APP/PS1 mice.

### YXQN Decreases Brain Aβ Levels by Altering APP Process in APP/PS1 Mice

**FIGURE 6:**
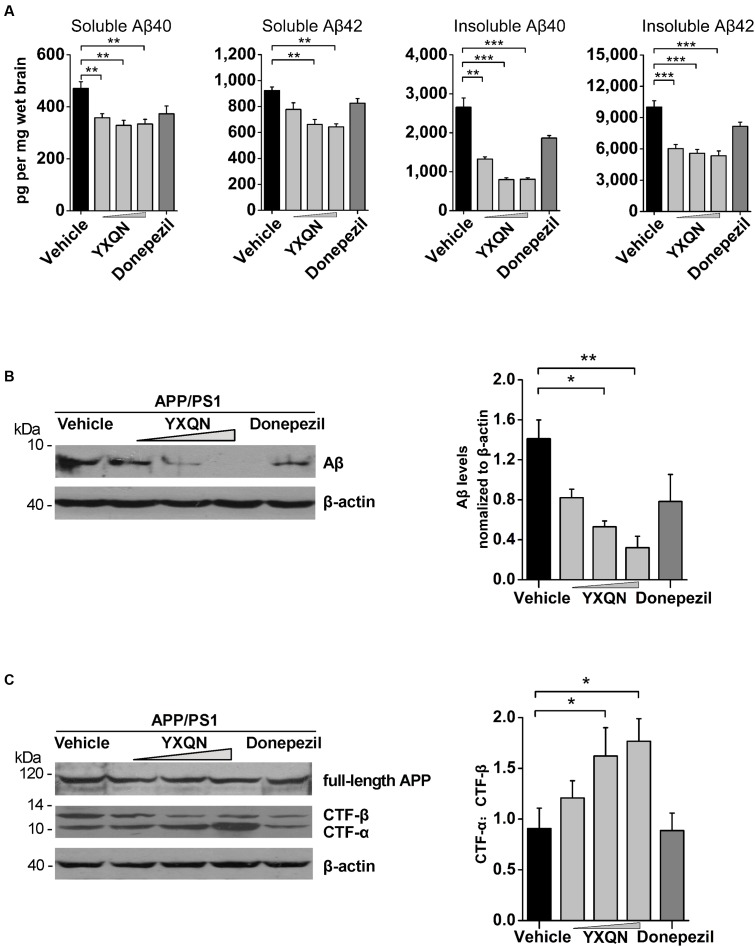
**Effects of YXQN on cerebral Aβ levels and APP process in APP/PS1 mice.** **(A)** The levels of formic acid-soluble (insoluble) and SDS-soluble (soluble) Aβ40 and Aβ42 in brain homogenates from YXQN administration from 8- to 10-month old using ELISA kits (*n* = 16–18). **(B)** The levels of total Aβ in the RIPA brain extracts of 10-month-old APP/PS1 mice analyzed by Western blot with antibody 6E10 (β-actin as internal controls). **(C)** The expression of full-length APP and carboxyl-terminal fragments (CTFs) analyzed by immunoblot with the APPct antibody and densitometric scanning, and quantified by ratio of C-terminal α-cleavage product (CTF-α) and β-cleavage product (CTF-β). Western blot images and densitometric quantification of blots is from at least three independent experiments (*n* = 6 mice per group). The drug dosages from low to high of YXQN extract are 0.69, 2.08, and 6.24 g/kg. The numbers presented are mean ± S.E.M. (Dunnet’s *post hoc* analysis). *^∗^p* < 0.05, *^∗∗^p* < 0.01, and *^∗∗∗^p* < 0.0001.

Accumulating evidence indicates that soluble Aβ oligomers and Aβ fibrils participate in the pathological and cognitive symptoms of AD through different processes (Larson and Lesne, 2012; Zahs and Ashe, 2013). To define which kind of Aβ assemblies YXQN is involved in, we examined the amount of Aβ40 and Aβ42 in the soluble (SDS-soluble) or in the insoluble (formic acid-soluble) fractions in cerebral homogenate by ELISA. The majority of extracellular aggregations of Aβ40 and Aβ42 were detected in the formic acid-soluble fractions. Of the two, Aβ42 is the more amyloidogenic form of the peptide, due to its more hydrophobic nature (Kayed et al., 2003; Sadigh-Eteghad et al., 2015). As shown in **Figure 6A**, compared with the vehicle group, there was a significant decrease in the levels of soluble Aβ40 (*F* = 9.069; *p* = 0.008, *p* = 0.001, and *p* = 0.001) and Aβ42 (*F* = 15.569; *p* < 0.0001 and *p* < 0.0001) in the YXQN middle- and high-dose groups. Moreover, compared with the vehicle group, the APP/PS1 mice in YXQN groups showed a 50–70% reduction in highly aggregated forms (insoluble, formic acid extract) both of Aβ40 (*F* = 43.774; *p* = 0.001, *p* < 0.0001, and *p* < 0.0001) and Aβ42 (*F* = 19.671; *p* < 0.0001, *p* < 0.0001, and *p* < 0.0001).

To further confirm the above results of ELISA that YXQN reduced the levels of Aβ, the amount of total cerebral Aβ were tested by Western blot. As shown in **Figure 6B**, the low-, middle-, and high-dose YXQN dramatically reduced the amount of Aβ in the brain, by 42, 62, and 77%, respectively, relative to the vehicle group (*F* = 6.339; *p* = 0.011, *p* = 0.003). To investigate APP processing involved in the reduction of Aβ levels, we further analyzed the expression of full-length APP and APP-derived C-terminal fragments (CTFα and CTFβ) in the brain of APP/PS1 mice in each group by immunoblot with the specific antibody. CTFα, one of the α-secretase-derived fragments from the non-amyloidogenic processing of APP, is a physiological product. Meanwhile, β-secretase-derived CTFβ from the amyloidogenic processing of APP is the pathologic product that further generates Aβ. As shown in **Figure 6C**, full-length APP was unchanged among different treatment groups. However, in YXQN treated APP/PS1 mice, the levels of CTFβ were remarkably decreased, along with an obvious increase of CTFα. Notably, the ratio of CTFα to CTFβ in the YXQN middle- and high-dose groups showed a 1.6∼1.8-fold elevation above that of the vehicle group by quantification (*F* = 3.593; *p* = 0.039 and *p* = 0.017). Thus, the results indicate a modulation effect of YXQN on suppressing amyloidogenic and promoting non-amyloidogenic processing of APP.

### YXQN Improves sAPPα Production by Promoting α-Secretase Expression

**FIGURE 7:**
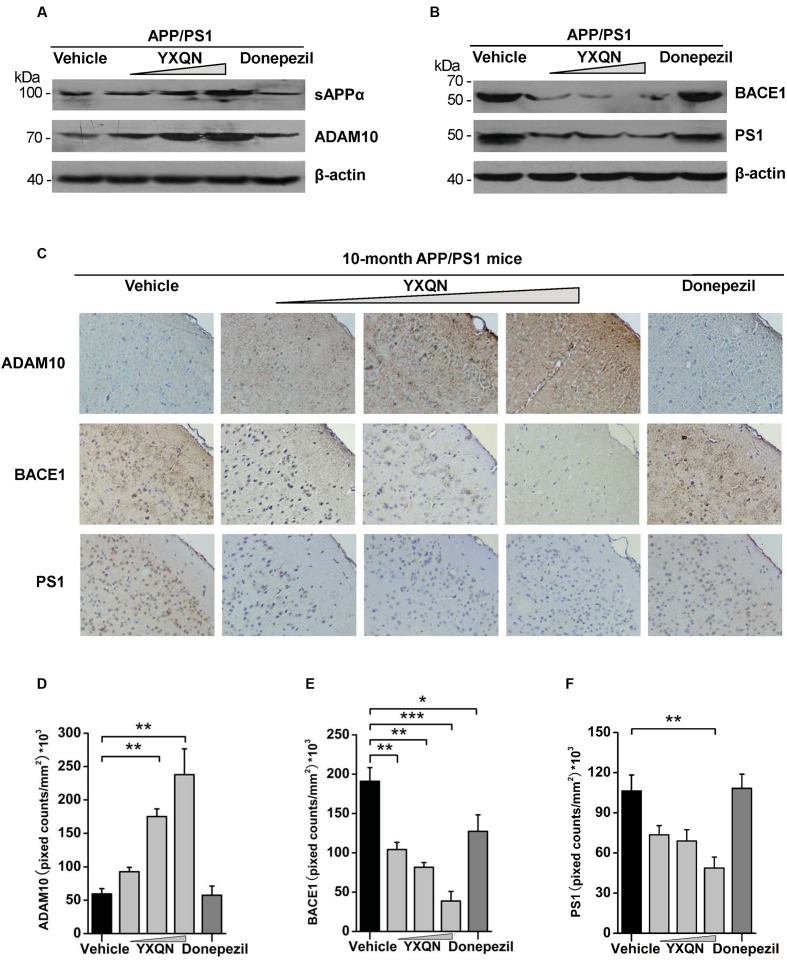
**The action of YXQN activates sAPPα and ADAM10, and inhibits BACE1 and PS1 expression demonstrated by Western blot and immunohistochemistry.** **(A)** Immunoblot analysis of α-cleavage product sAPPα and α-secretase (ADAM10) in the brain homogenates of 10-month-old APP/PS1 mice with vehicle, YXQN or donepezil administration. **(B)** BACE1 and PS1 from mouse brains were subjected to immunoblot analysis. Western blot images of at least three independent experiments are shown (*n* = 6 mice per group). **(C)** Staining for ADAM10, BACE1, and PS1 using specific antibody in sagittal sections of brain, and quantified by pixed counts (*n* = 6). The drug dosages from low to high of YXQN extract are 0.69, 2.08, and 6.24 g/kg. Mean ± S.E.M. ANOVA with Dunnet’s *post hoc* analysis was used **(D–F)**. *^∗^p* < 0.05, *^∗∗^p <* 0.01, and *^∗∗∗^p* < 0.0001.

Subsequently, to corroborate the effect of YXQN extract on non-amyloidogenic processing, we examined the expression of N-terminal α-secretase-derived sAPPα in cerebral hemispheres of each group, which has been reported as antagonizing amyloidogenic processing of APP and playing a neurotrophic role. Our results showed that the expression of sAPPα in YXQN middle- and high-dose groups presented a robust augmentation compared with the vehicle group (**Figure 7A**), which was consistent with the increase of CTFα. Given that ADAM10 as a common α-secretase cleaves APP to generate sAPPα and CTFα, the expression of ADAM10 in each group was detected by immunoblot. As shown in **Figure 7A**, a similar notable augmentation of ADAM10 was observed in YXQN middle- and high-dose groups, compared with the vehicle group. Then, we immunostained brain slices of the five groups with ADAM10 antibody and analyzed the total staining area. As shown in **Figure 7C**, compared with the vehicle group, the degree of ADAM10 staining in the cortex of YXQN middle- and high-dose groups strikingly increased. There was a meaningful difference between middle- or high-dose YXQN and vehicle APP/PS1 mice in the total positive stain cover area of ADAM10 (*F* = 16.433; *p* = 0.006 and *p* < 0.0001) (**Figure 7D**), which is in accordance with the results from the Western blot (**Figure 7A**). These data provided evidence that YXQN improved non-amyloidogenic processing of APP by promoting ADAM10 expression.

### YXQN Inhibits APP Pathological Process by Reducing BACE1 and PS1 Expression

BACE1 and PS1 as β- and γ-secretase play a core role in the pathological processing of APP (amyloidogenic processing) (Oddo et al., 2004; Willem et al., 2015). Based on the effects of YXQN in reducing the production of CTFβ and Aβ, the expression of BACE1 and PS1 were further detected in the brain of each group by both Western blot and immunohistochemistry, to confirm the roles of YXQN in the inhibition of APP pathological processing. As shown in **Figure 7B**, compared with the vehicle group, the expressions of BACE1 and PS1 in the brain tissues of YXQN groups were greatly reduced in a dose-dependent manner. A reduction of the BACE1 level was observed in the donepezil group as well. Moreover, in the analysis of the staining area of BACE1 and PS1 by immunohistochemistry, we found the BACE1 and PS1 levels markedly reduced in all three YXQN dosage level groups (**Figure 7C**). The total staining areas of BACE1 in low-, middle-, and high-dose groups were substantially decreased by 45, 57, and 79%, relative to the vehicle group (*F* = 15.754; *p* = 0.005, *p* = 0.001, and *p* < 0.0001); and that in the donepezil group also showed a slight reduction (*F* = 15.754; *p* = 0.032) (**Figure 7E**). Likewise, the three groups of YXQN, respectively, showed a 31, 45, and 54% reduction of PS1 expression in APP/PS1 mice, compared with vehicle APP/PS1 mice (*F* = 7.506; *p* = 0.005) (**Figure 7F**), suggesting the diminished production of Aβ peptide. Taken together, these data might indicate that YXQN extract inhibited the expression of BACE1 and PS1, and leaded to the reduction of Aβ in the brain of APP/PS1 mice. These results therefore addressed the suppression of amyloidogenic pathological processing of APP by YXQN.

All in all, our results confirm a significant role of YXQN against cognitive decline and Aβ aggregation in AD, through up-regulating the level of α-secretase ADAM10 in the physiological processing of APP, and down-regulating β-secretase BACE1 and γ-secretase PS1 in the pathological processing of APP.

## Discussion

Designing drugs to protect neurons from AD is very challenging, and large numbers of therapeutic drugs focusing on reducing Aβ levels have failed (Liu et al., 2014; Wisniewski and Goni, 2015). However, more and more clinical data show that the causes of AD are closely associated with CBF and brain nourishment. One traditional Chinese medicine, YXQN is a formula based on a famous decoction, Siwu Tang, which promotes blood circulation to alleviate headaches and dizziness, and which has been used clinically for 20 years. Our results demonstrated the pronounced effects of YXQN extract, not only on ameliorating cognitive and memory impairment, but also on mitigating the critical pathology in APP/PS1 mice.

A classical AD model, APP/PS1 mice were used in the present research. These mice were structured based on Aβ pathology, learning-memory deficit accompanied with detectable cerebral Aβ at 4 months (Jankowsky et al., 2004; Perez et al., 2005), significant amyloidosis at 6–7 months, and further aggravated at 10 months. Hence, 8-month APP/PS1 mice were administrated with diluted YXQN extract for 2 months, when the mice normally presented severe cognitive deficits and significant Aβ deposits, equivalent to moderate to severe AD patients. Excitingly, after 2 months of YXQN administration, YXQN APP/PS1 mice showed a substantial decrease in Aβ levels, compared to vehicle or donepezil APP/PS1 mice, in which the high-dose group showed a 47–72% reduction in plaque deposits relative to the vehicle group (**Figures 4**, **5**). While donepezil has been used widely to improve symptoms of AD, it functions not by Aβ-dependent pathogenic mechanisms, but by inhibiting cholinesterase. The significant Aβ reduction using YXQN compared to donepezil, revealed its specific attenuation of the Aβ deposition.

We focused on the morphometric analyses of decreased Aβ aggregation by YXQN in the CC-EC-hippocampus system. As a central part of the limbic system, the CC is closely associated with emotion formation and processing, learning, and memory (Frankland et al., 2004). As the key connection between the hippocampus and neocortex, the EC plays an essential role in spatial memories, including memory formation and consolidation (Lopez et al., 2014). The CC receives signals from the thalamus and the neocortex and sends them to the EC via the cingulum. The EC is one of the first regions of the brain to suffer from Alzheimer’s disease, with significantly decreased volume (Khan et al., 2014). Responsible for central spatial memory and navigation, the damage of the hippocampus is associated with memory loss and disorientation in AD (Takeda and Tamano, 2014; Chang et al., 2016). Our data shows that YXQN extract, especially in high doses, markedly reduces Aβ deposition in the AD associated regions, CC-EC-hippocampus system, accompanied with the ameliorative effect on spatial learning memory. The behavioral assessments, including MWM tests and Y-maze spontaneous alternation, showed that in a dose-dependent manner, YXQN extract substantially improved both long and short term spatial memory. The middle-dose of our experiments (2.08 g/kg for mouse) is equal with the clinical dose for patients (0.168 g/kg for human, about 10 g/per day), where there is certain clinical evidence of cognitive improvement with YXQN; in 60 patients with amnestic mild cognitive impairment (aMCI), the memory decline was delayed by a 3-month per oral course of YXQN, while in 35 patients with senile dementia it occurred by a 2-month course (Wang et al., 2005; Zhao et al., 2007).

The aggregation and deposition of Aβ is a foremost causative factor in AD pathogenesis. The cleavage of APP in both amyloidogenic or non-amyloidogenic pathways is regulated by three secretases, α-secretase (ADAM10) for the non-amyloidogenic processing of APP to CTFα and sAPPα, β-secretase (BACE1) and γ-secretase (PS1 mainly) to generate the Aβ fragment (Hoey et al., 2009; Portelius et al., 2011). Importantly, sAPPα shows an antagonistic action on amyloidogenic processing (Obregon et al., 2012; Tyan et al., 2012; Bailey et al., 2013). Our Western blot and immunohistochemistry results proved that YXQN increased the levels of CTFα and sAPPα by the higher expression of ADAM10 (**Figures 6C**, **7**), to play a neurotropic function against amyloidosis formation in AD mice. Further, the augmented ADAM10 were confirmed by the 6 times induction of mRNA transcriptional level (data not shown).

Because β- and γ-secretase are responsible for the amyloidogenic pathological processing of APP to generate Aβ fragments, altering their activity will change the production of Aβ (Esler and Wolfe, 2001; Oddo et al., 2004). Lower expressions of β- and γ-secretase could reduce the β-pathology process (Dewachter and Van Leuven, 2002; Willem et al., 2015). In this light, investigating the impact of YXQN extract on BACE1 and PS1 is of great interest. Western blot and immunohistochemistry verified that YXQN presented a dose-dependent inhibition of BACE1 and PS1expressions (**Figures 7B–F**), thus inhibiting the β-pathology process. We found YXQN decreased BACE1 through the inhibition of mRNA transcriptional level, but PS1 reduction may be associated with degradation pathway (data not shown). More importantly, unlike the direct inhibitors of β- or γ- secretase *in vitro*, YXQN reduced the levels of BACE1 and PS1 without altering the average lifespan and athletic ability of APP/PS1 mice. In short, YXQN significantly decreases amyloid plaques through two major aspects of the molecular mechanism: up-regulating the level of α-secretase ADAM10 in the physiological processing of APP, and down-regulating β-secretase BACE1 in the pathological processing of APP.

In addition to seven other Chinese medicines, the chief compounds of YXQN are Angelica sinensis, Ligusticum chuanxiong hort, White peony root, and Prepared radix rehmanniae, which have been used clinically as Siwu Tang for replenishing, nourishing, and increasing blood flow from as early as the Song dynasty (1000 years ago). Studies suggest that their probable molecular mechanism on AD treatment includes activating the neurotrophin signaling pathway and increasing CBF. The structure and function of neurons is maintained by the release of trophic factors. A new APP knock-in mouse model proved a direct and positive link between vascular and parenchymal Aβ, both of which can be modulated by CBF (Li et al., 2014; Sun et al., 2016). Findings indicate that decreased CBF might have implications for aMCI (Jefferson et al., 2015; Zamolodchikov et al., 2016). Another study reports that both amyloid plaques and decreased CBF are primarily associated with deficits in cognitive function (McDade et al., 2014). Moreover, neurotrophin related proteins such as the brain-derived neurotrophic factor have a protective role against Aβ toxicity (Lim et al., 2015). Using whole-genome DNA microarray to compare YXQN treated and vehicle APP/PS1 mice, we found 7 differentially expressed genes in the neurotrophin signaling pathway (data not shown). It appears that the 11 compounds of YXQN perform the functions of stimulating blood flow, anti-oxidation, protection of neurons, and regulation of the enzymes targeting the nervous system (**Table 1**); in which increasing CBF may relate to the effects of YXQN on enhancing ADAM10 and sAPPα; the anti-oxidation, neuroprotection and enzyme regulation may be associated with the effects of YXQN on inhibiting BACE1 and PS1, and activating ADAM10. Interestingly, neuronal overexpression of ADAM10 in transgenic mice reduces BACE1 processing of APP and amyloid deposition, that means the up-regulated α-secretase could promote anti-amyloidogenic processing of APP (Postina et al., 2004). In addition, our research about the effects of each component of YXQN on AD pathology related events in SH-SY5Y cell line presented that Angelicae sinensis, Ligusticum chuanxiong hort, Uncaria and Spatholobus suberectus were responsible for the increased sAPPα and decreased Aβ. Of them, Spatholobus suberectus also significantly increased the expression of ADAM10. While, the block of BACE1 expression was associated with Prunella Vulgaris or Cassia seed treatment. Further study is required to clarify the relative contribution of the individual ingredient of YXQN to the effects observed both *in vitro* and *in vivo*.

To date, YXQN has been commonly used for improving headaches, dizziness, giddiness, irritability and insomnia over 20 years. As YXQN formulated on the theoretical and clinical foundations of traditional Chinese medicine, it is effective toward multiple targets, especially, against the cognitive impairment caused by various cerebral vessel-related lesions. Data from 273 patients with chronic cerebral vascular insufficiency (CCI) at 9 hospitals in China demonstrated that, after 8 weeks of treatment, YXQN was as effective as nimodipine in improving the symptoms of CCI, including baseline in severity of headache, heavy-headed feeling, dizziness and sleep disorder (Wu et al., 2013); likewise, research in 83 patients showed that 12-week clinical YXQN treatment could effectively improve the CCI symptoms by reducing the vertigo score, and increasing middle cerebral artery mean velocity and vertebral artery mean velocity (Gu et al., 2005). Further, animal experiments also provided another line of evidence that YXQN increased CBF and attenuated cerebral microcirculatory disturbance in the ischemia-reperfusion injury, and therefore elevated model group rats’ memory performance (Gu et al., 2005; Xu et al., 2009; Xiong et al., 2011). In all, YXQN as a clinical medicine for vascular diseases by improving CBF is definite. Moreover, our work provides direct evidence on YXQN counteracts cognitive decline and decreases Aβ aggregation in AD mouse model. Overview, these studies indicate the multiple protective functions of YXQN on CBF associated disease, including CCI and AD.

In summary, our data provides lines of evidence that YXQN extract plays remarkably effective roles in reducing amyloid plaques in the brain, and in improving the cognitive decline of AD. In addition, at the clinical dose of YXQN and donepezil, YXQN shows more significant effect than the cholinesterase inhibitor, donepezil, on improving the cognitive decline of APP/PS1 mice by 2 months administration. Moreover, YXQN transfers APP processing from amyloidogenic to non-amyloidogenic by the activation of ADAM10 to enhance sAPPα levels. We have shown that YXQN could be a safe prospective anti-AD therapy directly addressing Aβ-dependent pathogenic mechanisms.

## Author Contributions

Conceived and designed the experiments: XW, RS, WL, ZL, LW, and XL. Acquired data: XW, RS, and ZL. Analyzed and interpreted the data: XW, XZ, YL, ZS, JL, and XL. Wrote the manuscript: XW and XL.

## Conflict of Interest Statement

The authors declare that the research was conducted in the absence of any commercial or financial relationships that could be construed as a potential conflict of interest.
